# Prevalence of Dental Implant Positioning Errors: A Radiographic Analysis

**DOI:** 10.3390/jcm14093221

**Published:** 2025-05-06

**Authors:** Razan Alaqeely, Abdullah Albaiz, Bassam Alenazi, Mohammed Alem, Yasser Alotaibi, Raed Alrowis

**Affiliations:** 1 Department of Periodontics and Community Dentistry, College of Dentistry, King Saud University, Riyadh 11545, Saudi Arabia; 2 Dental Intern, College of Dentistry, King Saud University, Riyadh 11545, Saudi Arabia

**Keywords:** implant positioning, cone-beam computed tomography (CBCT), implantology complications, peri-implant bone loss

## Abstract

**Objectives** Implant placement errors remain a persistent challenge, leading to complications such as peri-implant bone loss, neurosensory issues, and, in severe cases, implant failure. This study evaluates the prevalence and characteristics of dental implant positioning errors in patients treated at the Dental University Hospital. **Methods**: A sample of 500 cone-beam computed tomography (CBCT) scans was used to assess implants for positioning errors, including thread exposure, proximity to anatomical structures, and violations of inter-implant and implant–tooth distances. **Results:** A total of 56.6% of the implants exhibited positioning errors, with the maxillary posterior region being the most commonly affected area (51.6%). The most frequent errors observed were thread exposure (37.7%) and implant proximity to the maxillary sinus (27.7%). Statistical analysis revealed significant correlations between implant positioning errors and anatomical location, underscoring the need for meticulous preoperative planning and advanced imaging. While factors such as patient age, implant length, and diameter were analyzed, no statistically significant differences were found in error prevalence based on sex or demographic variables. **Conclusions:** This study highlights the importance of combining clinical expertise with advanced imaging modalities like CBCT to minimize implant positioning errors and improve patient outcomes. Future research should focus on refining surgical techniques and evaluating the impact of the implants’ design and patient-specific factors on the accuracy of placements.

## 1. Introduction

Edentulism, the condition of toothlessness, significantly affects individuals’ well-being and quality of life [1], despite a global decline in prevalence [2]. The prevalence of edentulism varies globally. In the United States, data from 2012 to 2020 indicate a significant downward trend in edentulism among adults aged 65 and older, decreasing from 16.36% to 13.54%. Conversely, in low- and middle-income countries (LMICs), edentulism remains a public health concern, with prevalence rates ranging from 2.9% to 15.3% [3]. In Saudi Arabia, a high percentage of adults had one or more missing teeth, with complete edentulism at 55 years and older [4]. This disparity underscores the need for targeted public health interventions to address the burden of edentulism in these regions.

Recent studies have highlighted that edentulism adversely affects various aspects of health, including frailty, general health, mental health, and mortality. For instance, a systematic review encompassing studies from high-income countries (HICs) and low- and middle-income countries (LMICs) found that edentulism negatively influences these health outcomes across diverse populations [5].

As life expectancy increases, the demand for rehabilitative treatments has grown, contributing to a larger senior population [6]. Dental implants, which integrate with the jawbone or skull to support dental prostheses such as crowns, bridges, and dentures, have become a cornerstone of modern dentistry for treating complete and partial edentulism. While dental implants are associated with safety [7], predictability, and a high success rate exceeding 90%, complications during placement remain a significant challenge for dental surgeons [8,9].

The diagnostic phase is crucial for the long-term success of dental implants, especially in selecting the appropriate radiographic examination. Panoramic radiography is the most suitable method for initial imaging assessments, often complemented by periapical radiography. The American Academy of Oral and Maxillofacial Radiology (AAOMR) recommends cross-sectional imaging for the evaluation of the implant site during the preoperative diagnostic phase. It also advocates for CBCT imaging as the preferred cross-sectional technique due to its high diagnostic output and reasonable radiation risk [10]. CBCT is particularly valuable for detecting implant positioning errors. It is recommended for patients with clinical symptoms such as discomfort and implant mobility, as it provides more detailed information than two-dimensional images [11]. Recent evidence supports its role in minimizing surgical complications and enhancing clinical outcomes. For instance, a 2022 study demonstrated that the anterior loop of the mental nerve was identified in 57% of cases using CBCT compared to only 17% with panoramic imaging, emphasizing the importance of CBCT in preventing nerve injury [12]. Furthermore, CBCT facilitates a precise dimension and density assessment of bone, enabling optimal implant positioning. A 2021 meta-analysis reported significantly lower implant failure rates (~2.3%) with CBCT-guided surgery compared to conventional freehand placement (~6.4%), underscoring the critical value of CBCT-based planning in reducing implant-related complications [13].

Technical errors may occur without producing early symptoms, underscoring the importance of CBCT imaging for the early identification of complications before clinical symptoms arise [9,14]. Implant positioning errors should not be confused with implant failure. Studies report implant failure rates between 2.9% and 7.2%. The implant type, patient age, and presence of a prosthesis may influence positioning errors. The improper angulation of dental implants can lead to unfavorable cosmetic outcomes, peri-implant bone loss, and implant failure [2,15]. While the maxillary arch, particularly its posterior region, is associated with complications such as sinus perforation and implant instability due to poor bone quality [16,17], improper positioning in the anterior maxilla can also affect esthetic outcomes and the thickness of keratinized tissue [18,19]. The bone is dense in the mandible compared to the maxilla, yet the risks are more significant in terms of causing injuries to vital neuromuscular structures [20,21]. Due to these anatomical factors, improper planning and surgical management might cause an improper distance between the implant and adjacent teeth. Such a malpositioning may result in hypersensitivity and masticatory discomfort and can contribute to implant thread exposure or horizontal alveolar bone resorption [15,22].

Misplaced dental implants can result in severe consequences, such as airway obstruction, bleeding, and implant loss due to a lack of osseointegration. These problems also lengthen the healing period, increase treatment costs, and cause discomfort for the patient and the dentist [10,23]. Previous data on the prevalence of dental implant positioning errors are scarce. Two studies conducted in Brazil found around 70–80% of errors, where thread exposure and an inadequate distance between implants and adjacent structures were the most common errors [15,24]. In Saudi Arabia, one study performed a clinical and radiographic analysis of 180 dental implants with positioning errors, which found the posterior maxilla as the most common area for errors [25]. However, the study included patients with signs and symptoms of complications.

To our knowledge, no study in Saudi Arabia has retrospectively examined incidental positioning errors on CBCT scans. This study aims to assess the prevalence of dental implant positioning errors and determine the most often affected oral locations in patients with dental implants.

## 2. Materials and Methods

This retrospective cross-sectional study utilized patient records from the College of Dentistry at King Saud University (KSU)’s electronic filing system. Ethical approval was obtained from the College of Dentistry Research Center (CDRC) and the Institutional Review Board (IRB) of the College of Medicine, King Saud University Medical City, Riyadh, Saudi Arabia. Patient confidentiality was ensured by assigning unique codes for analysis.

Cone-beam computed tomography (CBCT) scans of patients aged 18 and older treated at KSU DUH were examined. All CBCT scans acquired from July to December between 2018 and 2023 for various dental indications were considered, including but not limited to implant planning, endodontic evaluations, surgical planning, and orthodontic assessments. Scans with complete dentition, obscured implant views, or a poor image quality were excluded. All the included scans were obtained using a Planmeca ProMax 3D Plus unit (Planmeca Co., Helsinki, Finland) with parameters of 90 kV and 5–11 mA and a voxel size of 200 μm.

A comprehensive set of data points was extracted from each included CBCT file. This included patient demographics such as age at the time of the scan, gender, and nationality. Implant-related data included implant location, count, shape, length, diameter, and positioning errors. The evaluation was based on CBCT images in sagittal, axial, and coronal planes.

**Figure 1:**
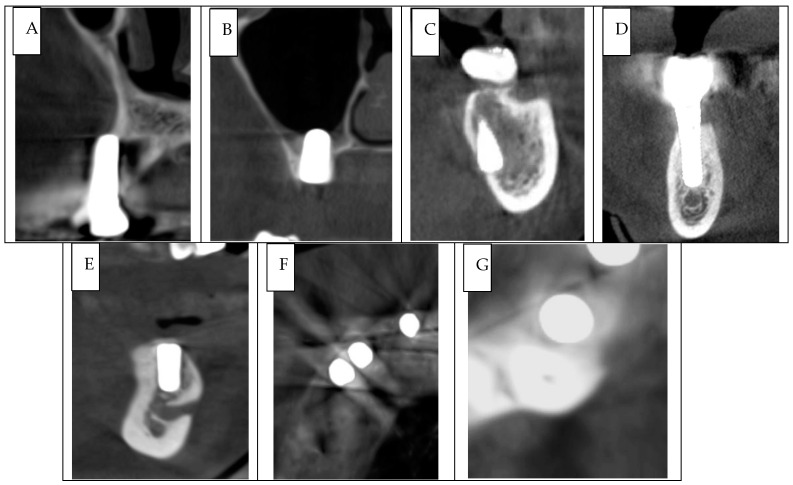
Cross-sectional images from CBCT scans illustrating implant malpositioning errors. (**A**) Cross-sectional image shows a thread exposure in a dental implant placed in the upper maxillary molars region. (**B**) Cross-sectional image shows a dental implant positioned inside the maxillary sinus. (**C**) Cross-sectional image shows a dental implant penetrating the cortical plate of the mandibular fossa. (**D**) Cross-sectional image shows a dental implant positioned in close proximity to the mandibular canal. (**E**) Cross-sectional image shows a dental implant positioned in close proximity to the mental foramen. (**F**) Cross-sectional image shows a violation of the minimum space between 2 dental implants. (**G**) Cross-sectional image shows a violation of the minimum space between the dental implant and a natural tooth.

The following criteria were used to define implant malposition:

- Thread Exposure: Bone loss exceeding 2 mm and inadequate bone support around the implant [9] (Figure 1A);
- Maxillary Sinus Penetration: Implant penetration into the maxillary sinus by at least 1 mm [11] (Figure 1B);
- Nasal Cavity Penetration: Implant penetration into the nasal cavity by at least 1 mm [11];
- Nasopalatine Foramen Contact: Implant proximity to the nasopalatine foramen within 2 mm;
- Mandibular Fossa Penetration: Implant penetration into the mandibular fossa (Figure 1C);
- Mandibular Canal Contact: Implant proximity to the mandibular canal within 2 mm [8] (Figure 1D);
- Mental Foramen Canal Contact: Implant proximity to the mandibular foramen (Figure 1E);
- Inadequate Inter-implant Distance: Distance between implants less than 3 mm (Figure 1F);
- Inadequate Implant-to-Tooth Distance: Distance between the implant and adjacent tooth less than 1.5 mm [26] (Figure 1G).

Four examiners (A.B., B.E., M.A., Y.O.) independently evaluated all the scans. All the evaluations were conducted in a darkened room. Patients were categorized into four age groups: <40 years, 40–59 years, 60–79 years, and ≥80 years.

### Statistical Analysis

The extracted data were initially organized and entered into an Excel spreadsheet, facilitating efficient data management and organization. Subsequently, the data were transferred to the Statistical Package for the Social Sciences (SPSS) 30 software for comprehensive statistical analysis. This study’s primary outcome, the prevalence of implant malposition, was determined through descriptive statistics. Chi-square tests explored potential associations between implant malposition and various demographic and clinical factors. Specifically, these tests assessed correlations between implant malposition and patient age, gender, the side of the implant’s placement (left or right), the type of positioning error (angular, linear, or rotational), and the jaw location (maxilla or mandible). Statistical significance was predetermined at a *p*-value of less than 0.05, indicating a 95% confidence level. Inter-rater reliability was analyzed using Cohen’s kappa test.

## 3. Results

Inter-rater reliability showed a good agreement between raters (0.8). After applying the inclusion and exclusion criteria, 500 CBCT scans were eligible. The CBCT scans were reviewed, of which 159 implants from 183 patients showed one of the positioning errors. Of these, 83 patients (45.4%) were male, and 100 (54.6%) were female. The upper posterior region exhibited the highest prevalence of errors (n = 82, 51.6%), followed by the lower posterior (n = 41, 25.8%), upper anterior (n = 24, 15.1%), and lower anterior (n = 12, 7.5%) regions. The lower posterior region had the highest percentage of correctly placed implants (n = 58, 47.5%), potentially due to the density and stability of the mandibular bone.

Thread exposure was the most frequent error observed in 60 implants (37.7%), particularly in the upper posterior region. This was followed by implant approximation to the maxillary sinus (n = 44, 27.7%), inter-implant distance violations (n = 29, 18.2%), and implant-to-tooth spacing violations (n = 15, 9.4%). Less common errors included implant penetration into anatomical structures, such as the nasal cavity (n = 3, 1.9%), mandibular fossa (n = 3, 1.9%), the mandibular canal (n = 4, 2.5%), and mental foramen (n = 1, 0.6%) (Table 1).

Gender showed no statistically significant association with the prevalence of positioning errors. Similarly, the implants’ diameter (mean 3.9 mm) and length (mean 9.8 mm) were not significantly correlated with the occurrence of errors. However, a significant correlation (*p* < 0.05) was found between implant positioning errors and anatomical location, with posterior regions showing a higher frequency of errors.

**Figure 2:**
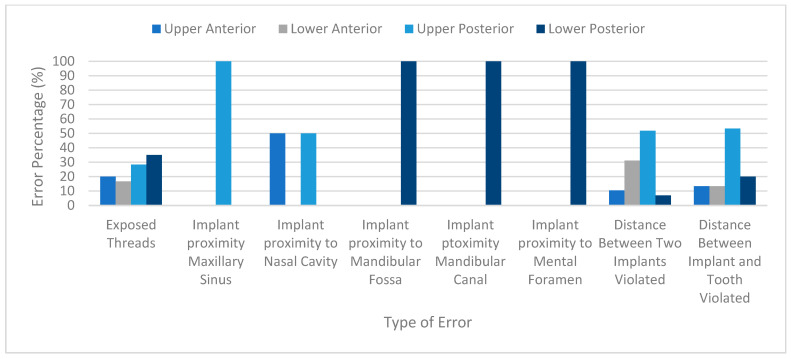
Implant positioning errors and their percentage of occurrence by region in the oral cavity.

Further analysis revealed regional variations in error types. For instance, maxillary sinus approximation was primarily observed in the upper posterior region (n = 41, 34.4%), while inter-implant distance violations were more common in the lower anterior region (n = 9, 30%). Figure 2 shows the distribution of positioning errors among the regions.

## 4. Discussion

This study highlights the prevalence and distribution of dental implant positioning errors, offering critical insights into potential areas of improvement in implantology. Our findings revealed a substantial proportion of implants with positioning errors (56.6%), which is lower than the prevalence reported in some previous studies, such as Ribas et al. (82.9%) and Rizzo et al. (74.4%) [15,24]. These discrepancies may stem from methodological differences, variations in population characteristics, or differing evaluation criteria.

Interestingly, no significant differences were observed in error prevalence based on patient demographics, including gender and implant dimensions. However, the high error rates in older patients (60–79 years) reflect the increased edentulism and demand for implants in this age group. These results align with those of Safi et al., who noted similar trends [23]. While our study did not extensively investigate the influence of implant design on positioning accuracy, other research suggests that factors such as the implant’s length and diameter could play a role, warranting further exploration of these variables across diverse populations [9,23].

Consistent with the prior research [15,24], the highest frequency of positioning errors (51.6%) was observed in the maxillary posterior region, particularly those involving thread exposure and proximity to the maxillary sinus. Similar to Ribas et al., our study demonstrated a statistically significant correlation between anatomical location and the type of positioning error. This regional variability emphasizes the need for customized treatment planning to address the unique challenges of different anatomical sites [15].

This emphasizes the importance of meticulous preoperative planning and the utilization of CBCT imaging to minimize risks, particularly in the posterior maxilla, where factors like atrophic ridges, sinus pneumatization, and bone quality can increase the likelihood of complications [27,28,29].

The predominance of thread exposure as the most prevalent error, followed by maxillary sinus approximation, aligns with observations from several studies [15,24,30]. This high occurrence may be attributed to the reduced bone density characteristic of the posterior maxilla, which can increase the likelihood of such errors [31,32]. Implementing conservative drilling techniques has been suggested to improve surgical outcomes in these scenarios [1,11].

Furthermore, Ribbas et al. discovered considerable error rates in the maxilla, particularly concerning violations of the necessary spacing between adjacent implants and teeth [15]. The present study also found significant violations of inter-implant distance (10.3% of total implants), with the highest rates in the lower anterior (30%) and upper posterior areas. The disparities in the prevalence of inter-implant distance violations between studies could be due to differences in implant design, surgical technique, or error assessment methods.

The implications of implant positioning errors extend beyond functional and esthetic outcomes. Addressing these errors can enhance patient-centered outcomes, such as an improved quality of life and reduced recovery times. Properly placed implants ensure better osseointegration, minimizing complications like peri-implantitis and implant failure, leading to quicker healing and a reduced need for additional interventions [10]. Moreover, accurate placement helps avoid neurosensory disturbances and functional impairments, enabling patients to regain chewing efficiency and speech clarity effectively. These improvements translate into higher patient satisfaction and greater confidence in oral rehabilitation [33]. From a broader perspective, reducing errors lowers treatment costs and psychological stress, further contributing to an improved quality of life for patients. Additionally, implants placed too close to anatomical structures, such as the maxillary sinus or mandibular canal, can lead to severe complications, including sinus perforation, chronic pain, and neurosensory deficits [34].

The findings of this study might provide some recommendations for improvements, despite the low number of included cases. To minimize common errors such as thread exposure and proximity to vital structures, clinicians should emphasize comprehensive preoperative planning, supported by advanced imaging techniques like CBCT. CBCT offers high-resolution, three-dimensional images that facilitate an accurate assessment of bone structures and identification of potential anatomical challenges, thereby aiding in precise implant placement. Standardized guidelines for interpreting CBCT scans can help identify site-specific challenges, particularly in anatomically complex regions like the posterior maxilla [1,11]. Another limitation of this study is that the operator information was not included in the data due to the difficulty of obtaining detailed information about the operators through hospital data from this sample. A higher number of CBCT scans and detailed information about the surgeon’s specialty and experience would provide more information about the effects of the operator factor on clinical placement errors and outcomes.

Furthermore, adopting digital workflows and surgical guides might be beneficial, as they can significantly enhance the placement accuracy by reducing the reliance on freehand techniques [35]. For instance, computer-aided design and manufacturing (CAD/CAM) technology can facilitate the creation of customized surgical templates tailored to each patient’s unique anatomy, improving precision and reducing complications [33]. The role of clinician expertise and continued education in minimizing errors cannot be overstated. A recent randomized clinical trial of guided implant placement for non-experts did not prevent complications, such as bone remodeling, compared to experts’ free-hand implant placement [35]. Knowledge, proper pre-operative planning, and surgical techniques are fundamentals to practicing the placement of dental implants [36].

Our recommendations for future research focus on long-term studies evaluating the impact of positioning errors on implant survival and patient-reported outcomes. Additionally, studies assessing the interventions’ clinical and economic impacts may offer insights into optimizing diagnostic protocols and reducing implant positioning errors. Long-term studies evaluating the impact of positioning errors on implant survival and patient-reported outcomes are warranted to guide best practices in implantology.

## 5. Conclusions

This study confirms a high prevalence of implant positioning errors, particularly in the posterior maxilla and, least of all, in the anterior mandible. These findings underscore the importance of meticulous planning and execution, especially in challenging anatomical areas such as the maxillary sinus and mandibular canal. Advanced imaging technologies like CBCT, combined with clinical expertise, can significantly mitigate the complications associated with implant placement. Further research is necessary to explore the influence of implants’ design, surgical techniques, and patient-specific factors on the accuracy of implant positioning.

## Figures and Tables

**Table 1:** Implant positioning error frequencies.

Implant Positioning Error	Frequency (n)	Percent (%)
Exposed Threads	60	37.73
Implant Approximation to Maxillary Sinus	44	27.67
Implant Approximation to With Nasal Cavity	3	1.89
Implant Approximation to Mandibular Fossa	3	1.89
Implant Approximation to Mandibular Canal	4	2.51
Implant Approximation to Mental Foramen	1	0.62
Distance Between Two Implants Violated	29	18.23
Distance Between Implant and Tooth Violated	15	9.43
Total	159	100

## Data Availability

Data are available upon request from the corresponding author.

## Abbreviations

The following abbreviations are used in this manuscript:

CBCT	Cone-Beam Computed Tomography
AAOMR	American Academy of Oral and Maxillofacial Radiology
CAD/CAM	Computer-Aided Design and Manufacturing
